# Advancements in noninvasive visualization of the immune environment in glioblastoma: A systematic review

**DOI:** 10.1093/noajnl/vdaf176

**Published:** 2025-08-04

**Authors:** Philipp Lohmann, Laura Schäfer, Sandra Krause, Betül Altunay, Antje Willuweit, Jan-Michael Werner, Norbert Galldiks, Karl-Josef Langen, Felix M Mottaghy, Susanne Lütje

**Affiliations:** Institute of Neuroscience and Medicine (INM-3, INM-4), Research Center Juelich, Juelich, Germany; Department of Nuclear Medicine, RWTH Aachen University Hospital, Aachen, Germany; Department of Nuclear Medicine, RWTH Aachen University Hospital, Aachen, Germany; Institute of Neuroscience and Medicine (INM-3, INM-4), Research Center Juelich, Juelich, Germany; Department of Nuclear Medicine, RWTH Aachen University Hospital, Aachen, Germany; Institute of Neuroscience and Medicine (INM-3, INM-4), Research Center Juelich, Juelich, Germany; Department of Neurology, Faculty of Medicine and University Hospital Cologne, University of Cologne, Cologne, Germany; Department of Neurology, Faculty of Medicine and University Hospital Cologne, University of Cologne, Cologne, Germany; Institute of Neuroscience and Medicine (INM-3, INM-4), Research Center Juelich, Juelich, Germany; Institute of Neuroscience and Medicine (INM-3, INM-4), Research Center Juelich, Juelich, Germany; Department of Nuclear Medicine, RWTH Aachen University Hospital, Aachen, Germany; Department of Radiology and Nuclear Medicine, Maastricht University Medical Center, Maastricht, The Netherlands; Center for Integrated Oncology (CIO), University Hospital RWTH Aachen, Aachen, Germany; Department of Nuclear Medicine, RWTH Aachen University Hospital, Aachen, Germany; Center for Integrated Oncology (CIO), University Hospital RWTH Aachen, Aachen, Germany; Department of Nuclear Medicine, RWTH Aachen University Hospital, Aachen, Germany

**Keywords:** brain tumors, glioma, immuno-PET, immunotherapy, immune imaging

## Abstract

**Background:**

Glioblastoma is known for its highly immunosuppressive microenvironment, hindering the efficacy of immunotherapies. Noninvasive imaging like immuno-positron emission tomography (PET) offers the potential for visualizing immune dynamics within glioblastoma, potentially aiding in patient selection and treatment monitoring. This systematic review evaluates immuno-PET tracers currently under investigation for the noninvasive visualization of the immune environment in glioblastoma.

**Methods:**

A literature search was conducted in PubMed and Web of Science up to March 2025, using keywords related to glioblastoma, immuno-PET, immune compartments, and specific tracers. Studies were screened based on predefined inclusion and exclusion criteria, focusing on the development, characterization, or application of immuno-PET tracers targeting immune cells or immune checkpoint molecules in glioblastoma.

**Results:**

Nineteen studies met the inclusion criteria, exploring tracers targeting immune checkpoints and immune cell populations. Full-length antibodies demonstrated higher tumor specificity and retention compared to smaller fragments but showed longer circulation times. Peptide-based tracers and affibodies offered improved pharmacokinetics with rapid clearance and lower nonspecific uptake but encountered hurdles in ensuring adequate tumor targeting and retention. Advancements included dual-modal tracers combining PET and near-infrared fluorescence imaging for enhanced diagnostic and intraoperative applications.

**Conclusions:**

Significant progress has been made in developing immuno-PET tracers for noninvasive visualization of immune reactions in glioblastoma. Challenges persist in clinical translation due to issues like blood–brain barrier permeability and safety profiles. Continued research and clinical evaluations are essential to harness the potential of immuno-PET in improving glioblastoma diagnosis, assessment of treatment response, and guiding personalized immunotherapy strategies, ultimately aiming to enhance patient outcomes.

**Key Points**

- Immune environment imaging may guide personalized immunotherapy in glioblastoma.
- Current PET tracers address nonoverlapping aspects of tumor–immune interactions.
- Imaging of distinct immune targets may enhance patient risk stratification.

**Importance of the Study**

Significant progress has been made in developing immuno-PET tracers for noninvasive visualization of immune reactions in glioblastoma. Overall, the breadth of immuno-PET tracers now being investigated—ranging from PD-L1-directed agents to those targeting CD8, CD4, or CD11b—addresses critical, nonoverlapping aspects of tumor–immune interactions. By merging advanced tracer design with improved blood–brain-barrier-disrupting technologies, noninvasive imaging of distinct immune targets may enhance nuanced patient risk stratification and selection. In addition, immune compartment visualization with PET imaging may guide treatment response evaluation and help to adapt personalized immunotherapeutic regimens to maximize efficacy in this complex and refractory disease.

Glioblastoma is the most aggressive primary brain tumor in adults, known for its rapid growth, high invasiveness, and dismal prognosis, with median overall survival times remaining below two years despite the implementation of aggressive multimodal treatment, including maximal safe surgical resection and radiotherapy with concomitant and adjuvant chemotherapy with alkylating agents.^1^ In addition to strong clinical prognostic factors such as age, Karnofsky-Index, and residual tumor remnants after surgery, the poor prognosis for patients with glioblastoma is also attributed to the tumor’s highly immunosuppressive microenvironment, being characterized by the absence of tumor-infiltrating lymphocytes, exhaustion of cytotoxic T-lymphocytes as well as high levels of immunosuppressive cytokines.^2^ This not only facilitates immune evasion but also presents substantial barriers to the efficacy of emerging immunotherapeutic approaches.^3,4^ While immunotherapies such as immune checkpoint inhibition have revolutionized treatment paradigms in various noncentral nervous system malignancies by enhancing T-cell mediated antitumor responses,^5^ clinical responses in brain metastases and glioblastoma remain limited.^6–9^ In randomized glioblastoma trials, adjuvant antiprogrammed cell death protein 1 (PD-1) administered in combination with radiotherapy after maximal safe resection and followed by anti-PD-1 monotherapy for 12 months, showed no benefit over the standard Stupp protocol.^6–8^ Neoadjuvant administration of immune checkpoint inhibition has been evaluated using single-agent anti-PD-1 in a small number of heavily pretreated patients with recurrent glioblastoma, showing that neoadjuvant blockade promotes the activation of tumor-infiltrating lymphocytes and extends overall survival.^10–12^ Recently, triplet immune checkpoint inhibition using nivolumab (anti-PD-1), ipilimumab (anticytotoxic T-lymphocyte protein 4 [CTLA-4]), and relatlimab (antilymphocyte-activation gene 3) was described in a case study of a newly diagnosed patient with glioblastoma, which suggested that neoadjuvant combination of immune checkpoint inhibition can promote infiltration, activation and expansion of tumor-specific T-cells in newly diagnosed glioblastoma.^13^

Taken together, this underscores the necessity for a deeper understanding of immune compartment dynamics within the glioblastoma tumor microenvironment. Establishing techniques for noninvasive visualization of the immune environment in glioblastoma is crucial for developing effective immunotherapies, selecting suitable patient populations for these therapeutic regimens and thereby enhancing patient outcomes.

Noninvasive imaging modalities, particularly positron emission tomography (PET), have the potential to elucidate the complex interplay between immune cells and glioblastoma cells. Immuno-PET leverages radiolabeled antibodies, antibody fragments, and peptides to target specific immune markers, thereby enabling the real-time quantitative assessment of target expression levels, immune cell infiltration, activation, and distribution within tumors. A series of PET tracers is currently under development, each designed to visualize distinct aspects of target expression, immune environment, and response in glioblastoma. Moreover, advancements in tracer design, including the development of dually functionalized agents that combine PET imaging with near-infrared fluorescence capabilities, are enhancing the specificity and utility of immuno-PET in both diagnostic and intraoperative settings.^14^ Additionally, focused ultrasound-assisted delivery methods are being explored to enhance the permeability of the blood–brain barrier, thereby improving the targeting and accumulation of immuno-PET tracers within the brain tumor milieu.^15^

This systematic review aims to evaluate the immuno-PET tracers currently under investigation for the noninvasive visualization of the immune environment of glioblastoma. By categorizing tracers based on their molecular targets, structural formats, and functional capabilities, strengths, and limitations of each approach are reviewed, highlighting advancements in tracer design and delivery. Furthermore, it explores the translational potential of these tracers in clinical settings, discusses how improved visualization of immune compartments can aid patient selection, assess treatment responses, and ultimately contribute to the development of more effective, personalized immunotherapeutic strategies for patients with glioblastoma.

## Methods

### Search Strategy

A comprehensive literature search was conducted to identify studies relevant to the noninvasive visualization of immune status and reactions in glioblastoma PET tracers. The following electronic databases were systematically searched from inception until March 2025: PubMed and Web of Science. The search strategy employed a combination of Medical Subject Headings (MeSH) terms and keyword phrases related to glioblastoma, immuno-PET, immune compartments, and specific tracers. The primary search terms included “glioblastoma,” “glioma,” “PET,” “immune-PET,” “immuno-PET,” “immune compartments,” “imaging,” combinations thereof and specific tracer names identified in preliminary screenings. Boolean operators (AND, OR) were used to refine the search results. Additionally, reference lists of relevant articles were hand-searched to identify any supplementary studies not captured in the initial database search.

### Inclusion and Exclusion Criteria

Studies were included in the systematic review if they met the following criteria:

- Population: Preclinical (animal models) and clinical studies involving subjects with glioma/glioblastoma.
- Intervention: Utilization of PET tracers designed to visualize immune compartments or immune responses within glioblastoma.
- Outcomes: Studies reporting on the development, characterization, or application of immuno-PET tracers targeting immune cells (eg, T-cells) or immune checkpoint molecules (eg, PD-1, PD-L1) in the context of glioblastoma.
- Study Design: Experimental studies, including both in vitro and in vivo preclinical models, as well as clinical trials.

Studies were excluded if any of the following criteria were met:

- Non-English Language: Articles not published in English.
- Study Type: Reviews, editorials, commentaries, case reports, and conference abstracts.
- Population: Studies focusing on cancers other than glioblastoma.
- Intervention: Use of imaging modalities other than PET/CT or PET/MRI or tracers not specifically targeting immune compartments.
- Outcomes: Studies not reporting relevant imaging outcomes or lacking sufficient data on tracer performance in immune visualization.

### Study Selection

All articles retrieved from the initial search were exported into reference management software (EndNote), and duplicates were removed. Subsequently, titles and abstracts of the remaining articles were screened to assess their eligibility based on the inclusion and exclusion criteria. Studies that potentially met the criteria or had unclear relevance were subjected to full-text review. A PRISMA flow diagram was constructed to illustrate the study selection process, including the number of articles identified, screened, eligible, and included in the review, along with reasons for exclusion at each stage.

### Data Synthesis

Given the heterogeneity of the included studies in terms of imaging agents, cancer types, study designs, and outcome measures, a meta-analysis was not feasible. Instead, a narrative synthesis approach was adopted. The extracted data were organized thematically based on molecular targets of the tracers.

## Results

### Literature Selection

A comprehensive search yielded ten studies that met the inclusion criteria for this systematic review, encompassing both preclinical and clinical investigations of immuno-PET tracers for the noninvasive visualization of immune reactions in glioblastoma. These studies collectively explored a series of tracers targeting distinct immune compartments, including programmed death-ligand 1 (PD-L1), CD69, CD8, and TIGIT, utilizing diverse molecular formats such as full-length antibodies, antibody fragments, affibodies, peptides, and dual-modal conjugates.

### Visualizing Immune Target Expression on Tumor Cells

#### PD-L1 targeting tracers.

**Figure 1.**
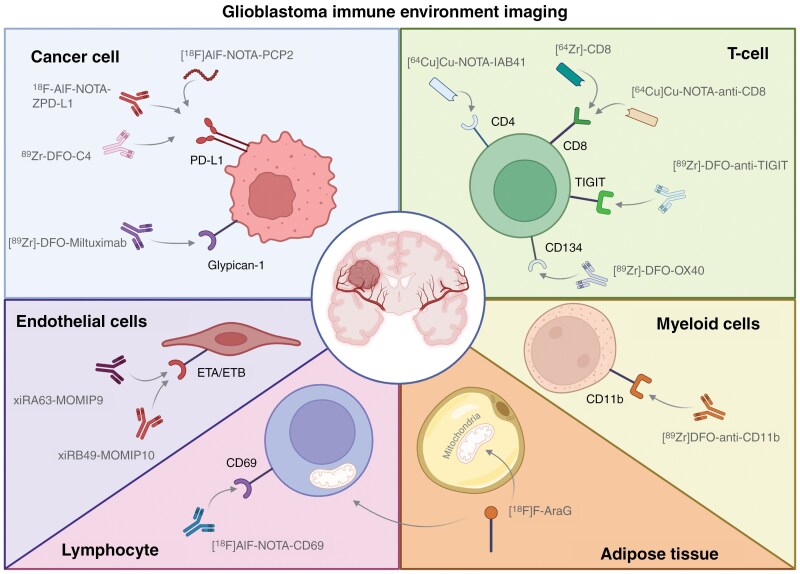
Schematic representation of novel imaging targets for visualization of glioblastoma immune environment.

—Recent advancements in molecular imaging have focused on improving the noninvasive visualization of PD-L1 in glioblastoma. PD-L1 is a critical immune checkpoint implicated in antitumor immune responses. High PD-L1 expression is associated with greater aggressiveness and invasiveness of glioblastoma cells and tumors with mesenchymal features seem to have increased PD-L1 levels and higher percentages of tumor-associated macrophages.^16^ Therefore, accurate measurement of PD-L1 expression seems critical for identifying/selecting patients suitable for anti-PD-(L)1 treatments. Here, six studies focused on imaging PD-L1 will be discussed.^15,17–21^ A schematic representation of the discussed tracers is provided in Figure 1.

Sharma et al. developed radiolabeled affibody molecules, ZPD-L1, using Fluorine-18 (^18^F) and Gallium-68 (^68^Ga) to target PD-L1 present in glioblastoma xenograft mouse models.^17^ These radioconjugates were synthesized with high radiochemical purity, and their binding affinity correlated with PD-L1 expression levels in vitro. In vivo imaging demonstrated that ^18^F-AlF-NOTA-Z_PD-L1_ effectively distinguished between PD-L1 high-expressing U87-MGvIII tumors and PD-L1-negative H292PD-L1KO tumors, providing high-contrast brain tumor images within one hour postinjection. Ex vivo biodistribution studies confirmed significant tumor uptake that correlated with immunohistochemical PD-L1 staining. Their findings suggest that anti-PD-L1 affibody-based immuno-PET imaging is a promising tool for glioblastoma patient stratification and could aid in the optimization of PD-1/PD-L1 checkpoint blockade therapy.

In a related effort, Chevaleyre et al. investigated the use of focused ultrasound to enhance the delivery of anti-PD-L1 antibodies across the blood–brain barrier and blood–tumor barrier for improved immuno-PET imaging. Native anti-PD-L1 IgG antibody (⁸⁹Zr-DFO-C4) was compared with a neonatal Fc receptor (FcRn) low-affinity mutant (⁸⁹Zr-DFO-C4^Fc-MUT^) in a murine syngeneic glioblastoma model.^15^ Transcranial focused ultrasound was applied before antibody injection to disrupt the blood–brain and blood–tumor barrier and facilitate antibody entry into the brain. The application of focused ultrasound significantly increased brain uptake of both antibody formats. Notably, the FcRn low-affinity mutant exhibited a shorter plasma half-life and reduced efflux from healthy brain tissue, enhancing tumor-specific binding earlier than the native antibody. Immuno-PET imaging showed that ⁸⁹Zr-DFO-C4^Fc-MUT^ provided optimal contrast in brain tumors at 22 h postinjection, compared to 7 days for the native antibody. This study highlights that engineering antibodies to reduce neonatal Fc receptor interactions, combined with focused ultrasound-induced blood–brain barrier disruption, can improve the quantitative imaging of PD-L1 expression in glioblastoma.

Zirconium-89 (^89^Zr)labeled atezolizumab (anti-PD-L1 antibody) was used to visualize the dynamic presence of PD-L1 in mice bearing orthotopic glioblastoma tumors and in patients with newly diagnosed glioblastoma.^21^ Immuno-PET specifically detected tumor and immune cell PD-L1, even after neoadjuvant Pembrolizumab. Preclinical studies confirm tracer uptake correlates with BBB disruption and PD-L1 expression. Clinical data reveal heterogeneous but distinct tracer accumulation in glioblastoma subregions, highlighting the potential of ^89^Zr-DFO-Atezolizumab for guiding personalized immunotherapy.

Advancing the development of novel imaging agents, Wang et al. introduced a peptide-based PET tracer, [^18^F]AlF-NOTA-PCP2, designed to enhance the imaging of PD-L1 heterogeneity in glioblastoma xenografts.^19^ The tracer was synthesized with high radiochemical purity and demonstrated strong and specific affinity for PD-L1, comparable to the established tracer [^18^F]AlF-NOTA-WL12. In vitro studies confirmed the tracer’s specificity, showing increased uptake in PD-L1-expressing cell lines and significant reduction in competitive binding assays. In vivo PET imaging effectively visualized PD-L1 levels and spatial heterogeneity, achieving higher tumor-to-blood ratios and lower nonspecific liver uptake compared to the existing tracer. Toxicity assessments indicated no significant abnormalities, highlighting the tracer’s safety profile. The improved pharmacokinetic properties, rapid renal clearance, and lower radiation exposure suggest that [^18^F]AlF-NOTA-PCP2 is a highly sensitive and specific tool for noninvasive quantification of PD-L1 levels, with significant potential for enhancing the precision of cancer immunotherapy.

Subsequently, dynamic PD-L1 expression changes in glioblastoma after radiotherapy could be detected using [^18^F]AlF-NOTA-PCP2 as a tracer. Fractionated regimens were suggested to induce stronger PD-L1 upregulation.^20^

Further exploring the applications of [^18^F]AlF-NOTA-PCP2, another study by Wang et al. evaluated its efficacy in visualizing PD-L1-driven radioresistance in glioblastoma.^18^ The tracer not only quantified the spatial heterogeneity of PD-L1-expressing cells but also showed increased uptake postradiotherapy in PD-L1-positive tumors, aligning with observed radioresistance. Mechanistic studies revealed that elevated PD-L1 contributes to radioresistance by enhancing DNA damage repair through the PI3K-Akt/RAD51 pathway, independent of its immune checkpoint functions. Additionally, preliminary clinical application in a glioblastoma patient demonstrated the tracer’s ability to safely and effectively monitor PD-L1 dynamics in a clinical setting. These findings indicate that [^18^F]AlF-NOTA-PCP2 offers a sensitive, specific, and safe method for noninvasive, real-time visualization of PD-L1-driven radioresistance in glioblastoma, supporting its potential role in guiding personalized radiotherapy and immunotherapy strategies.

Collectively, these studies underscore the significant progress in developing molecular imaging agents and techniques to noninvasively assess PD-L1 levels in glioblastoma. While affibody molecules like ^18^F-AlF-NOTA-Z_PD-L1_ offer rapid imaging capabilities with high contrast, peptide-based tracers such as [^18^F]AlF-NOTA-PCP2 provide improved pharmacokinetics and lower nonspecific uptake. However, challenges remain in translating these tracers to clinical practice due to factors such as blood–brain barrier penetration, safety profiles, and regulatory approvals. These innovative approaches hold promise for improving patient stratification, guiding individualized therapeutic interventions, and ultimately enhancing the efficacy of immunotherapy and radiotherapy in glioblastoma.

### Visualizing Target Expression on Tumor-Infiltrating Immune Cells

While the studies mentioned above focus on visualization of an immune checkpoint present on tumor cells, it is also possible to image immune cells such as effector T-cells infiltrating tumor tissues. As these cells are key players in antitumor immune responses and tumor eradication, visualizing the infiltration of these cells into the tumor may be very valuable for therapeutic assessment.

#### CD8 targeting.

—Cytotoxic CD8-positive T-cells play an essential role in antitumor immune responses through the release of effector molecules including cytokines, perforin, and granzyme B.^22^ In addition, increased CD8-positive T-cell infiltration has been associated with positive treatment responses and improved prognosis in a series of preclinical studies.^23,24^ So far, the first data indicate the usefulness of CD8-targeting immuno-PET for tracking immune responses in several tumor entities.^25–27^

In brain tumors, Nagle et al. developed a ^64^Cu-labeled NOTA-anti-CD8 minibody tracer to image CD8-positive T-cells.^28^ In humanized mice bearing orthotopic glioblastoma xenografts, the tracer specifically accumulated in the spleen, lymph nodes, and tumor regions with dense CD8-positive T-cell infiltrates, surpassing background uptake in normal brain. Preclinical imaging and autoradiography confirmed a strong correlation between tracer signal and actual T-cell density.

**Figure 2.**
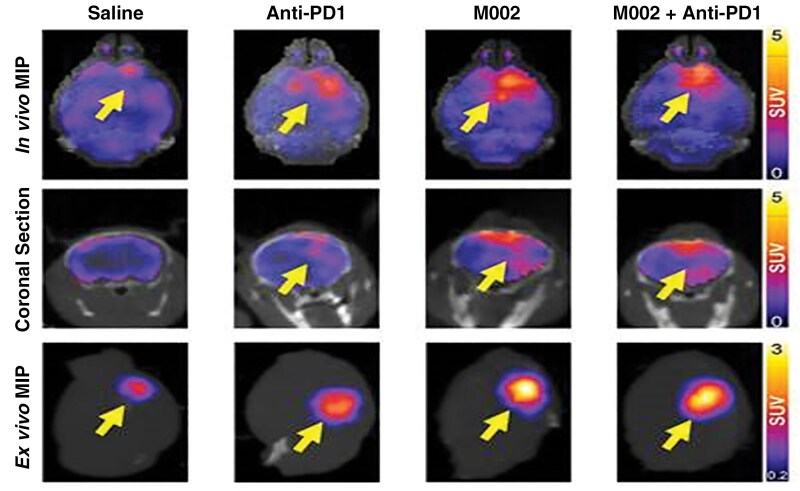
[^89^Zr]-CD8 immuno-PET imaging showing increased CD8 + T-cell infiltration in combination M002 and anti-PD-1 immunotherapy. Representative in vivo [^89^Zr]-CD8 minibody immunoPET brain MIP (top row), coronal head cross section (middle row), and ex vivo brain MIP (bottom row). Signal uptake can be seen in regions of tumor implantation as pointed by the yellow arrows on each representative image.^29^ This figure was adapted from Gallegos CA, Lu Y, Clements JC, Song PN, Lynch SE, Mascioni A, Jia F, Hartman YE, Massicano AVF, Houson HA, Lapi SE, Warram JM, Markert JM, Sorace AG. Theranostics. 2024 Jan 1;14(3):911-923.

Gallegos et al. evaluated [^89^Zr]-CD8 Immuno-PET imaging to monitor CD8-positive T-cell infiltration and predict survival outcomes in mouse models undergoing combination immunotherapy.^29^ Orthotopic syngeneic glioblastoma models treated with different therapies were utilized and longitudinal [^89^Zr]-CD8 PET imaging to quantify CD8-positive T-cell presence were conducted. Selective tumor accumulation was shown, being significantly higher in immunotherapy-treated tumors compared to controls (Figure 2). This uptake strongly correlated with increased CD8-positive cell density and improved survival in responders. Additionally, responders exhibited a more homogeneous distribution of CD8-positive cells within the tumor microenvironment, which was associated with enhanced therapeutic efficacy. These findings demonstrate that [^89^Zr]-CD8 immuno-PET is a highly sensitive and specific method for visualizing CD8-positive T-cell dynamics, providing valuable prognostic information and supporting its potential application in guiding personalized immunotherapy strategies for glioblastoma patients. However, the long circulation time of full-length antibodies like [^89^Zr]-CD8 may limit their clinical utility due to prolonged radiation exposure.

#### CD4 *targeting.*

—Compared to CD8-positive T-cells, the contribution of specific CD4-positive T-cell subsets in antitumor immune responses has not been characterized in detail so far.^30–32^ Notwithstanding, it has been shown that CD4-positive T-cells are necessary to maintain CD8-positive antitumor immune responses in mouse models.^33,34^ Clinical studies link CD4-positive T-cells to the development and maintenance of antitumor immunity.^35,36^ Upon activation, naïve CD4-positive T-cells differentiate into distinct subtypes, including immunosuppressive regulatory T-cells (Tregs) and helper cells capable of tumor lysis.^37,38^ Given the role of CD4-positive T-cells in PD-1/PD-L1 responses, there is an unmet need for noninvasive methods to track CD4-positive T-cells in brain tumors over time.

Nagle et al. evaluated the humanized anti-CD4 minibody, [^64^Cu]Cu-NOTA-IAB41, for noninvasive imaging of human CD4-positive T-cells.^30^ They showed that the tracer binds selectively to diverse CD4-positive T-cell populations without affecting T-cell proliferation or phenotype. In PBMC-humanized mice bearing orthotopic glioblastoma xenografts, [^64^Cu]Cu-NOTA-IAB41 effectively visualized CD4-positive tumor-infiltrating lymphocytes in the brain, correlating with autoradiographic and histological findings. Additionally, the tracer identified peripheral CD4-positive T-cell accumulations in the spleen and lungs. Overall, [^64^Cu]Cu-NOTA-IAB41 enables real-time, quantitative immuno-PET imaging of CD4-positive T-cell distribution in vivo, offering a promising approach for studying cancer immunotherapies and neuroinflammatory processes.

#### CD11b targeting.

—Glioblastomas are known to be heavily infiltrated by tumor-associated myeloid cells (TAMCs).^39^ As TAMCs are known to suppress antitumor immune responses and promote tumor cell growth, their quantification by noninvasive imaging could potentially facilitate patient selection for TAMC-targeted therapies as well as for the assessment of treatment response.^40^ The uniform expression of the cell surface marker integrin CD11b on TAMCs renders CD11b as a potential target for PET imaging.

Nigam et al. evaluated a ^89^Zr-labeled, DFO-chelated anti-CD11b antibody for immuno-PET to noninvasively assess TAMCs in a syngeneic orthotopic glioblastoma model.^40^ PET imaging and biodistribution revealed high tracer accumulation in the tumor-bearing hemisphere and spleen, correlating with elevated CD11b-positive cell infiltration. Flow cytometry confirmed significant CD11b receptor expression on myeloid cells in the tumor, while immunohistochemistry validated increased CD11b-positive cell density. Furthermore, blockade with a lower-specific-activity tracer markedly reduced uptake, demonstrating specificity. Overall, this approach accurately measures TAMCs in vivo, highlighting the potential of immuno-PETs to quantify immunosuppressive cell populations, guide patient selection, and monitor glioma immunotherapies.

#### CD69 targeting.

—CD69, a C-type lection protein, is one of the cell surface proteins expressed earliest by activated lymphocytes.^41^ Importantly, it is expressed by mature activated T-cells and platelets, and expression is not found in resting circulating leukocytes.^42^ CD69 is involved in lymphocyte proliferation and functions. Nisnboym et al. developed a novel peptide-based PET tracer, [^18^F]AlF-NOTA-CD69 antibody, designed for imaging T-cell activation by targeting CD69, an early T-cell activation marker.^43^ The tracer was synthesized with high specificity and affinity for CD69, confirmed through in vitro assays and molecular docking studies. In murine glioblastoma models undergoing immune checkpoint inhibitor therapy, [^18^F]AlF-NOTA-CD69 antibody selectively accumulated in tumors treated with immune checkpoint inhibitors, correlating strongly with increased CD69 expression on tumor-infiltrating lymphocytes. Higher tracer uptake post immune checkpoint inhibitor therapy was associated with increased T-cell activation and longer survival, establishing a positive correlation between CD69 immuno-PET signals and prognosis. Additionally, the tracer exhibited minimal nonspecific uptake in normal tissues, enhancing tumor-to-background contrast.

In addition, the potential of CD69 as a biomarker of ICI response in patients with glioblastoma was assessed. ScRNA-seq data from two published datasets of primary IDH-wild-type glioblastoma were analyzed, including datasets of a cohort of four newly diagnosed adult patients with glioblastoma and of 20 adults and 8 pediatric patients with glioblastoma.^44,45^ Together, these data support that CD69 can serve as a putative biomarker for monitoring changes of T-cell immune responses in the TME following ICI administration in patients with glioblastoma.^43^ Overall, the study concluded that CD69 immuno-PET holds promise in the assessment of immunotherapy efficacy and guiding personalized treatment strategies in glioblastoma.

#### CD134/OX40 *targeting.*

—CD134 (or OX40) is a co-stimulatory receptor of the TNF superfamily that is primarily expressed on activated effector T-cells and Tregs, while generally absent on naive and resting memory T-cells.^46,47^ It drives proliferation and survival of effector and memory T-cells, while inhibiting Treg development and function,^48^ thus enhancing antitumor responses. Anti-OX40 monoclonal antibodies have shown favorable outcomes in murine tumor models, including glioma.^49,50^ Vaccination further boosts these responses by inducing immunologic memory through strongly immunogenic stimuli such as CpG oligodeoxynucleotides (TLR9 agonists).^51,52^ Although local injection of CpG in glioblastoma proved limited, subcutaneous vaccine approaches incorporating tumor lysates have yielded systemic immunity and therapeutic benefits.^53,54^ Notably, repeated intradermal vaccinations with tumor lysate and CpG, followed by intraperitoneal injection of Fc-OX40L, significantly expanded brain-infiltrating CD4-positive and CD8-positive T-cells in murine glioma models, suggesting that integrating OX40 activation with targeted vaccination can strengthen glioblastoma immunotherapy.^55^

Nobashi and colleagues evaluated a ^89^Zr-labeled DFO-chelated OX40 monoclonal antibody PET tracer to track activated T-cells in a murine orthotopic glioma model.^56^ Immune-PET detected high OX40 levels in lymphoid organs (ie, axillary and cervical lymph nodes, spleen) and correlated with tumor regression. This approach provided whole-body assessment of T-cell responses, highlighting how remote vaccination can stimulate systemic immunity against glioblastoma. Their findings support the clinical potential of OX40/CD134-targeted immuno-PET to monitor and predict immunotherapy effectiveness in glioblastomas.

#### TIGIT targeting.

—Vincze et al. developed a novel immuno-PET tracer, [^89^Zr]-DFO-anti-TIGIT (^89^Zr-αTIGIT), to visualize TIGIT (T-cell immunoreceptor with Ig and ITIM domains) expression within the tumor microenvironment of glioblastoma.^57^ TIGIT is an immune checkpoint receptor present on activated T-cells and natural killer (NK) cells that suppresses immune responses by binding to ligands like CD155, inhibiting T-cell and NK-cell functions. The tracer was created by conjugating an anti-TIGIT antibody with a DFO chelator and radiolabeling it with Zirconium-89, achieving high radiochemical purity while maintaining its strong immunoreactivity, specifically binding to TIGIT-expressing cells in vitro. In vivo PET imaging and biodistribution studies in glioblastoma mouse models showed that ^89^Zr-αTIGIT accumulated within the tumor microenvironment, with specificity confirmed through blocking studies. However, tracer uptake in tumors was minimal compared to control tracers, highlighting limitations in its sensitivity and tumor visualization. Future optimization might involve modifying the tracer format, such as using smaller antibody fragments or alternative labeling methods, to enhance tumor penetration and improve imaging sensitivity. While the study demonstrated a proof-of-concept for specifically targeting TIGIT in the microenvironment of glioblastomas, further optimization for effective immuno-PET imaging and clinical applicability is necessary.

### Targeting Glypican-1

Glypican-1 (GPC-1) may play a role in modulating immune responses by influencing cell signaling pathways involved in tumor growth and immune evasion.^58–60^ As a cell surface heparan sulfate proteoglycan, GPC-1 can interact with growth factors and cytokines, and promote cell proliferation and inhibit apoptosis,^61,62^ potentially altering the tumor microenvironment and immune surveillance. Its high expression in various solid tumors, including glioblastoma, makes it a critical biomarker for both therapeutic targeting and diagnostic imaging.

Ghosh et al. evaluated [^89^Zr]-DFO-Miltuximab and its antibody fragments (Fab′₂, Fab, and scFv) as GPC-1 targeting immuno-PET agents for glioblastoma imaging.^63^ Miltuximab, a clinical-stage chimeric monoclonal antibody developed by GlyTherix Ltd., specifically targets GPC-1. Miltuximab and its fragments were radiolabeled with ^89^Zr and structural integrity and binding affinity to GPC-1 were confirmed through in vitro assays. In vitro studies demonstrated that all ^89^Zr-labeled formats were specifically internalized in GPC-1-expressing glioblastoma cell lines. However, the full-length Miltuximab exhibited the highest tumor retention and superior tumor-to-background ratios compared to the smaller fragments. In vivo PET/CT imaging in subcutaneous glioblastoma mouse models showed that [^89^Zr]-DFO-Miltuximab effectively accumulated and was retained within the tumor microenvironment. In contrast, the antibody fragments displayed increased uptake in the liver and kidneys, leading to reduced tumor specificity. These findings underscore the importance of antibody format and molecular size in designing effective immuno-PET tracers for glioblastoma.

### Targeting Neuroinflammation and Adipose Tissue Activation

Levi et al. investigated the relationship between neuroinflammation and adipose tissue activation using [^18^F]F-AraG, a mitochondrial metabolic tracer capable of tracking both activated lymphocytes and adipocytes.^64^ In murine models of glioblastoma and multiple sclerosis, [^18^F]F-AraG imaging revealed a significant correlation between intracerebral immune infiltration and activation of brown adipose tissue and bone marrow adipose tissue. This suggests that immune activation in glioblastoma could have systemic effects, influencing metabolic processes beyond the central nervous system. Notably, in clinical settings, postacute COVID-19 subjects exhibited concurrent [^18^F]F-AraG uptake in the brain and adipose depots, suggesting a neuroinflammation-adipose tissue link in humans. These findings propose an intricate immuno-neuro-adipose circuit, highlighting the potential of [^18^F]F-AraG as a dual-purpose tracer for visualizing immune and metabolic interactions within the glioblastoma microenvironment. Understanding this link may offer new insights into glioblastoma progression and open avenues for therapeutic interventions targeting systemic immune-metabolic pathways.

Another potential immune target is translocator protein (TSPO), a transmembrane protein located in the outer mitochondrial barrier. TSPO has been associated with functions in steroid synthesis, regulation of proliferation, apoptosis, and migration.^65^ Compared to other healthy tissues, TSPO is normally expressed at very low levels in the central nervous system.^66^ TSPO expression is upregulated at sites of inflammation or neurodegeneration and also in gliomas.^67^ Weidner and colleagues evaluated the association between the TSPO tracer [^18^F]GE180 uptake with expression of cell lineage markers by immunohistochemistry.^68^ Tracer uptake in PET was associated with TSPO immunohistochemistry, and while tumor core areas seemed to be a major contributor to the overall TSPO signal, TSPO signals in the tumor rim were mainly driven by CD68-positive microglia/macrophages. Molecularly, high TSPO expression seems to mark prognostically unfavorable glioblastoma cell subpopulations characterized by an enrichment of mesenchymal gene sets and higher amounts of tumor-associated macrophages.^68^

### Noninvasive Detection of Active Immune Niches in Skull Bone Marrow

The radioligand [^68^Ga]Ga-Pentixafor is directed to the C-X-C motif chemokine receptor 4 (CXCR4) protein that is enriched in hematopoietic and immune cell niches. [^68^Ga]Ga-Pentixafor PET imaging revealed pronounced radiotracer uptake in the cranial bone marrow adjacent to glioblastomas, a site previously considered immunologically inert in adults.^69^ This suggests immune cell activation in the calvarial marrow near the tumor at first diagnosis. Regions of high CXCR4 signal corresponded with increased presence of tumor-reactive CD8-positive effector memory T-cells in the proximal cranial bone marrow, a population characterized by durable antitumor potential. These cells shared clonotypes with tumor-infiltrating lymphocytes, suggesting recirculation and functional linkage between bone marrow and tumor. The detection of tumor-adjacent cranial bone marrow (functioning as a reservoir and active participant in antitumor immunity) via [^68^Ga]Ga-Pentixafor PET imaging challenges the traditional view of brain immune privilege. However, a more detailed investigation is needed to elucidate the actual antitumor effects mediated by T-cells originating from the cranial niche.

### Dual-Modal Tracers

One innovative study introduced dual-modal tracers combining PET and near-infrared fluorescence imaging to enhance glioblastoma detection and surgical precision.^14^ Antibody conjugates targeting endothelin receptors ETA and ETB were developed, which play significant roles in cancer progression and are promising therapeutic targets. Endothelin receptors are involved in tumor growth and survival as well as in modulating tumor angiogenesis and immune responses^70,71^; thus, targeting ETA and ETB receptors may also provide insights into the biological behavior of the tumor, immune responses, and potential therapeutic targets. The antibody conjugates were engineered with the IRDye800CW fluorophore and DFO for ^89^Zr chelation, enabling both PET and near-infrared fluorescence imaging capabilities. Utilizing click chemistry, they synthesized homogeneous constructs with high affinity and specificity for their targets. Preclinical validation in mice bearing ETA and ETB-positive tumors demonstrated effective accumulation of these tracers in endothelin receptor-positive glioblastomas. This might facilitate precise tumor detection, improving diagnostic accuracy and aiding fluorescence-guided surgery. The study underscores the versatility of dual-modal tracers targeting endothelin receptors in enhancing tumor detection specificity and surgical outcomes for patients with glioblastoma.

### Comparative Efficacy and Limitations

A summary of the reviewed PET tracers for noninvasive visualization of immune reactions in glioblastoma is provided in Table 1. Across the reviewed studies, full-length antibodies consistently demonstrated higher tumor specificity and retention compared to smaller fragments, albeit with increased nonspecific uptake in metabolically active organs such as the liver and kidneys. Tracers targeting early activation markers (CD69) and specific immune cell populations (CD8) showed promising correlations with therapeutic outcomes and survival, highlighting their potential utility in monitoring immunotherapy responses. On the other hand, tracers targeting immune checkpoints like TIGIT faced challenges in achieving sufficient tumor uptake and specificity, indicating the need for further optimization. Additionally, the development of dual-modal tracers presents a promising avenue for integrating diagnostic and surgical applications, though their complexity requires meticulous design to maintain functionality and specificity.

**Table 1.** Summary of PET Tracers for Noninvasive Visualization of Immune Reactions in Glioblastoma

Target	Radiotracer	Targeting agent	Clinical/preclinical	Tumor entity	Cell lines	Year	Reference
PD-L1	^18^F-AlF-NOTA-Z_PD-L1_ ^68^Ga-NOTA-Z_PD-L1_	Affibody	Preclinical	Nonsmall-cell lung cancer Glioblastoma	H292 H292_PD-L1KO_ U87-MGvIII GCGR-E55	2023	^17^
	^89^Zr-DFO-C4 ^89^Zr-DFO-C4^Fc-MUT^	Antibody	Preclinical	Glioblastoma	GL261-GFP	2023	^15^
	^89^Zr-DFO-Atezolizumab	Antibody	Preclinical + clinical	Glioblastoma	GL261 GL261_PDL1KO_ GCGR-E55 BL-6-NPE-IE	2024	^21^
	[^18^F]AlF-NOTA-PCP2 [^18^F]AlF-NOTA-WL12	Peptide	Preclinical	Glioblastoma	U87MG U87^PD − L1OE^ U87^PD − L1KO^ U251MG SF126 A172	2024	^19^
	[^18^F]AlF-NOTA-PCP2	Peptide	Preclinical	Glioblastoma	U87MG U251MG U118MG A172	2024	^20^
	[^18^F]AlF-NOTA-PCP2	Peptide	Preclinical + first-in-human pilot study	Glioblastoma	U87MG U87^PD − L1OE^ U87^PD − L1KO^ U251MG SF126 A172	2025	^18^
CD8	[^64^Cu]Cu-NOTA-anti-CD8	Minibody	Preclinical + normal human brain	Glioblastoma	PDX160721-1 PDX160615-1	2021	^28^
	[^89^Zr]-CD8	Minibody	Preclinical	Glioblastoma	GSC005-luc	2024	^29^
CD4	[^64^Cu]Cu-NOTA-IAB41	Minibody	Preclinical	Glioblastoma	PDX160721-1 PDX160615-1	2022	^30^
CD11b	[^89^Zr]DFO-anti-CD11b	Antibody	Preclinical	Glioma	GL261	2020	^40^
CD69	[^18^F]AlF-NOTA-CD69	Antibody	Preclinical + patient material	Glioblastoma	Jurkat T-cells (clone E6-1) GL261	2023	^43^
CD134	[^89^Zr]-DFO-OX40	Antibody	Preclinical	Glioma	GL26α8	2021	^56^
TIGIT	[^89^Zr]-DFO-anti-TIGIT	Antibody	Preclinical	Glioblastoma	GL261	2024	^57^
GPC-1	[^89^Zr]-DFO-Miltuximab	Antibody Fab′2 Fab scFv	Preclinical	Glioblastoma	U251	2023	^63^
Adipocytes	[^18^F]F-AraG	Guanosine derivative	Preclinical + clinical	Glioblastoma Postacute COVID-19	GL261-luc2	2024	^64^ NCT04815096 NCT02323893
TSPO	[^18^F]GE180	Ligand	Clinical	Glioblastoma	–	2023	^68^
Endothelin	xiRA63-MOMIP 9 xiRB49-MOMIP10	Antibody	Preclinical	Glioblastoma	CHO-ET_A_ CHO-ET_B_ CHO-WT	2023	^14^
CXCR4	[^68^Ga]Ga-Pentixafor	Peptide	Clinical	Glioblastoma	–	2024	^69^

## Discussion

This systematic review highlights substantial progress in the development of immuno-PET tracers designed for the noninvasive visualization of glioblastoma’s immune environment. While many studies have focused on different tracer formats—full-length antibodies, minibodies, peptides, affibodies—to quantify targets such as PD-L1, CD8, or CD69, the underlying rationale for imaging these varied immune pathways and cell types is also critical. noninvasive visualization of targets like PD-L1 (present on tumor and immune cells), CD4/CD8 (on T-cell subsets), CD11b (on myeloid-lineage cells), CD69 (on early activated T-cells), and OX40 or TIGIT (as co-stimulatory/co-inhibitory checkpoint molecules) provides a window into multiple facets of the glioma immune reaction, offering richer insights than tumor-centered imaging alone.

### Rationale for Imaging Different Immune Targets

Imaging immune checkpoints such as PD-L1 or TIGIT is particularly useful for determining whether and when tumors rely on these inhibitory pathways to evade cytotoxic T-lymphocytes. By contrast, visualizing effector T-cells themselves via CD8 or even CD4 can directly show the presence, distribution, and infiltration of these critical antitumor populations. In this context, imaging CD8-positive T-cells can help clinicians predict which glioblastomas are likely to respond to CD8-focused immunotherapeutics, while simultaneously ensuring that T-cell infiltration is robust enough to warrant treatments like immune checkpoint inhibitors. Visualizing CD4-positive T-cells, including helper and Treg populations, delivers complementary information about the broader immune landscape. As regulatory T-cells and helper T-cells exert profound effects on tumor immunity and therapy outcomes, noninvasive quantification of these subsets could enable early assessment of immune-mediated resistance or synergy with combination treatments.

Similarly, tracers like anti-CD11b enable the localization and quantification of TAMCs, which are a substantial immunosuppressive force in the glioblastoma microenvironment. Monitoring these cells noninvasively could inform interventions targeting myeloid-derived cells, a strategy that may enhance or modulate T-cell-centered therapies in patients. Additionally, imaging of early activation markers such as CD69 or co-stimulatory molecules such as OX40 can capture dynamic facets of the T-cell activation cycle and help tailor immunotherapy schedules. In essence, each immune marker addresses a different question:

- Do tumors overexpress inhibitory ligands like PD-L1?
- Are T-cells present and abundant (CD4/CD8)?
- Are myeloid cells dominating (CD11b)?
- Are T-cells fully activated (CD69/OX40)?

### Molecular Format Considerations

The reviewed studies underscore how full-length antibodies often provide high tumor retention and specific binding at the cost of longer circulation times, potentially higher off-target accumulation, and concerns regarding radiation dose. In parallel, smaller formats—including minibodies, affibodies, peptides—exhibit desirable pharmacokinetics, faster clearance, and enable earlier imaging time points, though they may exhibit shorter tumor retention or reduced affinity. For imaging complex immune processes, these differences matter significantly: short-lived tracers may capture acute immune changes (eg, transient expression of activation markers), whereas longer-lived antibodies can accumulate over time and highlight sustained immunological phenomena such as persistent checkpoint expression or myeloid infiltration.

### Enhancing Delivery to the Brain

Even optimized formats face limitations in assessing the typically heterogeneous blood–brain barrier integrity and accessing infiltrative tumor regions that are often protected by an intact blood–tumor barrier. Studies deploying focused ultrasound to disrupt these barriers, as showcased in a subset of articles, suggest that precisely timed barrier disruption can increase tracer uptake within glioblastoma tissue. This strategy can broaden the scope of immune markers imaged, as previously suboptimally distributed tracers (eg, large IgGs) may now enter tumor regions more effectively.

### Future Directions

Future investigations should expand beyond single-target imaging. Given that immune compromise in glioblastoma involves multiple cell populations—cytotoxic T-cells, helper T-cells, Tregs, tumor-associated macrophages, and others—parallel or multiplexed imaging of these various compartments could generate more comprehensive data. Such approaches could guide combination immunotherapies targeting different arms of the immune system (eg, checkpoint blockade plus macrophage depletion), revealing synergy or antagonism. In addition, systematically assessing how tracer binding changes over time throughout disease progression is crucial to accurately capture shifts in target expression and immune infiltration. Additionally, clinical trials evaluating these tracers in patients must consider safety profiles, scaling of tracer doses, and regulatory pathways. Verifying how well immuno-PET correlates with histopathological measurements of the same immune populations in resected samples will be key for validation. Finally, dual or even triple tracer protocols (eg, PD-L1 combined with CD8 or CD4 imaging) could offer more nuanced insight into the interactions among different immune pathways, potentially leading to improved patient stratification and treatment guidance.

## Conclusion

Overall, the breadth of immuno-PET tracers now being investigated—ranging from PD-L1-directed agents to those targeting CD8, CD4, or CD11b—addresses critical, nonoverlapping aspects of tumor–immune interactions. By merging advanced tracer design with improved BBB-disrupting technologies, noninvasive imaging of distinct immune targets may enhance more nuanced patient risk stratification, the assessment of treatment response, and help to adapt personalized immunotherapeutic regimens to maximize efficacy in this complex and refractory disease.
